# Dupsifter: a lightweight duplicate marking tool for whole genome bisulfite sequencing

**DOI:** 10.1093/bioinformatics/btad729

**Published:** 2023-12-13

**Authors:** Jacob Morrison, Wanding Zhou, Benjamin K Johnson, Hui Shen

**Affiliations:** Department of Epigenetics, Van Andel Institute, Grand Rapids, MI 49503, United States; Center for Computational and Genomic Medicine, Children’s Hospital of Philadelphia, Philadelphia, PA 19104, United States; Department of Pathology and Laboratory Medicine, University of Pennsylvania, Philadelphia, PA 19104, United States; Department of Epigenetics, Van Andel Institute, Grand Rapids, MI 49503, United States; Department of Epigenetics, Van Andel Institute, Grand Rapids, MI 49503, United States

## Abstract

**Summary:**

In whole genome sequencing data, polymerase chain reaction amplification results in duplicate DNA fragments coming from the same location in the genome. The process of preparing a whole genome bisulfite sequencing (WGBS) library, on the other hand, can create two DNA fragments from the same location that should not be considered duplicates. Currently, only one WGBS-aware duplicate marking tool exists. However, it only works with the output from a single tool, does not accept streaming input or output, and requires a substantial amount of memory relative to the input size. Dupsifter provides an aligner-agnostic duplicate marking tool that is lightweight, has streaming capabilities, and is memory efficient.

**Availability and implementation:**

Source code and binaries are freely available at https://github.com/huishenlab/dupsifter under the MIT license. Dupsifter is implemented in C and is supported on macOS and Linux.

## 1 Introduction

The presence of duplicate reads, where a read is an exact copy of another, can lead to biased results when performing whole genome sequencing (WGS) and whole genome bisulfite sequencing (WGBS) analyses (Smith *et al.* 2014, Rochette *et al.* 2023). When duplicate reads collect in locations in the genome, they can skew the results of counting experiments, where a precise number of unique reads is important for delineating between events, especially rare events. Duplicate reads primarily come in two varieties: polymerase chain reaction (PCR) and optical duplicates.

Current sequencing techniques generally work through binding individual DNA fragments in a solution to a flow cell or bead, amplifying the molecule into a cluster of fragments, then sequencing by synthesis of the cluster. The probability of binding a single fragment is dependent on the concentration of DNA. Therefore, most library preparation protocols include a PCR amplification step to increase the amount of input DNA (usually 1–100 nanograms for bulk libraries and around 5 picograms for single-cell libraries) to the microgram level, which increases the chance of each fragment being sequenced, at the cost of some fragments being sequenced more than once. PCR duplicates are the result of multiple copies created during amplification binding to the flow cell or bead and being sequenced. Because of the additional amplification needed for single-cell protocols, it is common to see higher fractions of PCR duplicates in single-cell experiments relative to bulk experiments. Optical duplicates, on the other hand, result from the sequencer separating a single cluster into two or more clusters, creating multiple duplicate reads.

**Figure 1.**
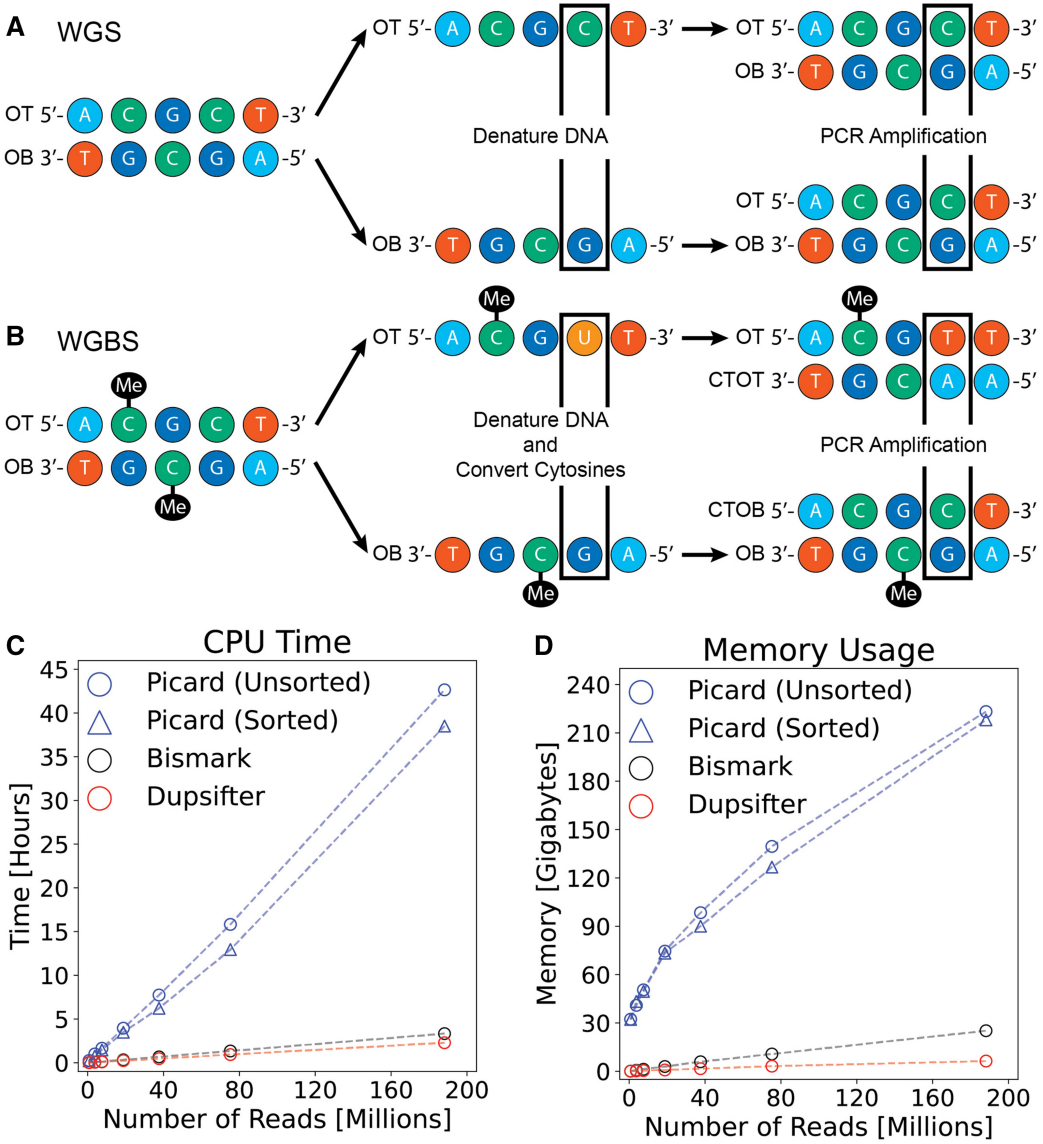
Dupsifter handles WGBS-related library preparation differences during duplicate marking and performs better than other alternatives. (A) Initial steps in performing PCR amplification in a WGS library preparation. (B) Initial steps in a WGBS library preparation for unmethylated cytosine conversion and PCR amplification. (C) CPU time for running Picard MarkDuplicates, Bismark’s deduplicate_bismark, and dupsifter. (D) Peak memory usage for Picard, deduplicate_bismark, and dupsifter.

PCR amplification is often used for WGS and WGBS library generation. However, there is one important difference in the DNA fragments that are created for WGS and WGBS (Fig. 1A and B) libraries. When PCR amplifying a WGS library, the process proceeds as shown (Fig. 1A), whereas a WGBS library has an extra step before PCR amplification is run. In this case, the DNA is denatured and sodium bisulfite is added to the solution, converting unmethylated cytosines into uracils, before running PCR amplification. This added step results in four distinct strands of DNA (Fig. 1B, Xi and Li 2009): one deriving from the original top (OT) strand, one from the original bottom (OB) strand, and two from the complements of each strand (CTOT and CTOB). This means there are two distinct copies of DNA for a given DNA fragment in WGBS, but only one distinct copy in WGS.

Single-cell sequencing approaches often rely on cell barcodes to multiplex many cells into a pool, primarily for throughput and to overcome sequencer input requirements when starting with a single cell. It also allows for profiling many cells simultaneously, as well as streamlined processing, as each cell is “tagged” with a unique barcode found within each alignment in an alignment file (e.g. BAM). In this context, duplicate marking cannot be done by naively identifying matching read starts and ends. Instead, the duplicate read marking tool must be aware of reads/fragments originating from multiple cells and not marking these reads as duplicates since they may represent distinct fragments across single cells.

Because of the possibility of biased results due to duplicate reads, several tools have been written to mark or remove duplicates from aligned data. For WGS data, tools such as samblaster (Faust and Hall 2014), picard MarkDuplicates (http://broadinstitute.github.io/picard/), samtools markdup (Danecek *et al.* 2021), and sambamba markdup (Tarasov *et al.* 2015) are widely used. However, because of the possibility of two original copies in WGBS, these tools are insufficient for distinguishing between two reads that are true PCR duplicates and one read that comes from the original top strand and one from the original bottom strand.

Bismark’s (Krueger and Andrews 2011) deduplicate_bismark is currently the only duplicate marking tool that correctly handles WGBS PCR duplicates. However, it only works for data aligned with Bismark and is unable to handle reads marked as secondary or supplementary or paired-end reads where both reads are unmapped. Furthermore, it cannot accept streamed input or stream the output. Deduplicate_bismark also uses a large amount of memory, especially for BAMs with one hundred million reads or more, which is not an uncommon depth for WGBS experiments. Dupsifter was written to provide a WGBS-aware duplicate marking tool that works with commonly used WGBS aligners, including Bismark, BISCUIT (https://github.com/huishenlab/biscuit), gemBS (Merkel *et al.* 2017), and bwa-meth (Pedersen *et al.* 2014). Dupsifter can accept streamed input, such as from BISCUIT or bwa-meth, as well as running with an already aligned BAM. Additionally, dupsifter is capable of streaming duplicate marked reads for use as input to other downstream tools, such as samtools for coordinate sorting. Dupsifter natively handles unmapped and nonprimary read alignments, which are included in the output from both BISCUIT and bwa-meth. By using a custom data structure for saving unique signatures as they occur, dupsifter is able to efficiently use memory, particularly when compared against deduplicate_bismark. Finally, dupsifter is able to mark duplicates in WGS data with minimal effort.

## 2 Description

Dupsifter is written in C and utilizes htslib (Bonfield *et al.* 2021) and klib’s hash implementation (https://github.com/attractivechaos/klib, specifically the khashl.h implementation). Htslib provides native reading and writing of SAM/BAM files, as well as accepting input from standard input and writing to standard output. Klib’s hash implementation is a lightweight, open-source hashing implementation that seamlessly integrates with dupsifter.

Similar to samblaster, dupsifter concatenates all contigs listed in the SAM/BAM file into a single “supercontig” before creating bins by splitting the supercontig into chunks of approximately the same size. By forming bins this way, dupsifter reduces the memory overhead for storing the available contigs, which is particularly helpful for large genomes, such as plants, or those with many contigs. Read positions can be calculated by finding the offset (a combination of the original contig lengths and padding added to account for read clipping during alignment) from the beginning of the supercontig.

In order to determine which strand a read derives from (OT/CTOT or OB/CTOB in Fig. 1B), dupsifter uses SAM auxiliary tags added to a read through the WGBS aligner of choice. If these tags do not exist, then a comparison of the number of C→T and G→A conversions is used to determine the strand. Dupsifter handles WGS data by assuming all reads derive from the same original strand.

The read signature in dupsifter is defined by the bin number and position of reads 1 and 2 (read 2 is a fixed bin number and position for all reads in single-end data), whether read 1 is to the left of read 2 (ignored for single-end data), the read orientation, whether the data is single-end or paired-end, and an optional barcode. By default, the barcode is fixed for all reads; however, for sequencing protocols with multiplexed barcoding (e.g. Luo *et al.* 2018), the barcode is taken, in order of priority, from the CB SAM tag, the CR SAM tag, or the read name. A duplicate is determined by coupling the signature with the strand the read(s) derive from. The first occurrence of a read is set as the nonduplicate, while all further occurrences are marked as a duplicate. Because of the possibility of secondary and supplementary alignments, dupsifter only uses the primary reads to determine if a read is a duplicate. That said, if a read is determined to be a duplicate, all primary, secondary, and supplementary alignments are marked as such.

Practically, dupsifter treats all duplicates that occur as a generic duplicate, and ignores the underlying cause. Therefore, PCR and optical duplicates are both handled by dupsifter.

## 3 Results

In order to show performance improvements of dupsifter over deduplicate_bismark, as well as Picard MarkDuplicates, which is commonly used for WGBS duplicate marking, time and peak memory usage data was collected across a wide range of input BAM sizes. Dupsifter is more efficient in its CPU usage (Fig. 1C), particularly compared to Picard. While the relative difference to deduplicate_bismark is more modest, the ability for dupsifter to handle streamed input and output means that end-to-end alignment times would be reduced for those analyses using dupsifter, as duplicate marking can be done simultaneously with alignment. In addition, wall time comparisons show modest improvement for dupsifter over the other tools (Supplementary Fig. S1). Further, dupsifter uses significantly less memory (Fig. 1D). Based on the memory usage calculated, if a user had sufficient memory to store the BAM and a moderate amount of RAM, a laptop could be used to mark duplicates with dupsifter.

In order to compare the results of marking duplicates with dupsifter and Picard MarkDuplicates, as well as the impact of marking duplicates of cytosine-converted reads with WGS tools, two single-cell extended representation bisulfite sequencing (scXRBS) replicates (Shareef *et al.* 2021) were aligned with BISCUIT and then duplicate marked. Because Picard uses a different signature for defining a potential duplicate and a different method for choosing the nonduplicate, as well as not properly accounting for WGBS, dupsifter was also compared against itself in nonbisulfite mode. This removes any differences in defining and selecting duplicates, thereby allowing a direct comparison of the impact of reads coming from the same location, but on different strands. When comparing dupsifter against itself, the fraction of reads incorrectly marked due to not properly accounting for bisulfite treatment is small (∼0.1% in both replicates, Table 1). On the other hand, the percentage of reads that were duplicate marked differently between dupsifter and Picard was much larger (∼15%, Table 1). This implies the differences between Picard and dupsifter are largely driven by differences in the definition and selection of duplicates rather than the fraction of reads from the same location, but on different strands. Despite these differences, the overall duplicate rate is broadly consistent between the two tools (<2% difference between them, Table 1).

**Table 1.** Comparison of duplicate rates between dupsifter in both bisulfite-aware (BS) and nonbisulfite-aware (non-BS) modes and Picard MarkDuplicates.^a^

Differences between Dupsifter (BS mode) and Dupsifter (non-BS mode)			
Duplicate	Not duplicate	Rep. 1 (%)	Rep. 2 (%)
BS	Non-BS	0.0	0.0
Non-BS	BS	0.123	0.103
Total Different		0.123	0.103

Differences between Dupsifter (BS mode) and Picard (non-BS)			
Duplicate	Not duplicate	Rep. 1 (%)	Rep. 2 (%)
Dupsifter	Picard	6.654	6.652
Picard	Dupsifter	8.584	8.51
Total different		15.238	15.162

Duplicate rate		
Duplicate marking tool	Rep. 1 (%)	Rep. 2 (%)
Dupsifter (BS mode)	22.394	23.606
Dupsifter (non-BS mode)	22.517	23.709
Picard (non-BS)	24.324	25.464

a (top) Comparison of reads with different duplicate status in dupsifter ran in BS and non-BS modes. (middle) Comparison of reads with different duplicate status in dupsifter and Picard MarkDuplicates. (bottom) The duplicate rate relative to the number of read pairs.

We also performed a small analysis to compare the relative impact of duplicate marking tool choice on methylation levels using the two scXRBS replicates compared previously and two GM12878 WGBS replicates (Dunham *et al.* 2012, Supplementary Fig. S2). Methylation levels were broadly consistent in the WGBS datasets with only 7%–10% of CpGs having a difference in methylation level >0.2. The scXRBS datasets were slightly higher with a methylation level difference of 11%–13%.

All performance benchmarking was performed on a server-class machine with 40 cores (2 Intel Xeon Gold 6138 CPUs running at 2.0 GHz per core) and 384 GB of memory. Details about how the benchmarking and duplicate marking comparisons were performed can be found in the Online Supplementary Information.

## 4 Discussion

In theory, duplicate marking with WGBS could allow for identifying hemi-methylation, or the presence of differing methylation states across both strands at a CpG locus. A statistical argument for the occurrence of hemi-methylation could be made by identifying reads that occur at the same location, but have different methylation states on the two strands. However, this is beyond the scope of this paper, as further work is needed to test the feasibility of such identification and to thoroughly evaluate the statistical argument.

Because of the additional step of denaturing the DNA and conversion of unmethylated cytosines in WGBS compared to WGS, a WGBS-aware duplicate marking tool is needed to correctly handle reads in a WGBS library. With respect to previous analyses that did not use a WGBS-aware duplicate marking tool, our brief comparisons of dupsifter against Picard MarkDuplicates show differences in duplicates rates and methylation levels that would not have substantial impacts on biological conclusions. However, due to the small sample sizes used in these comparisons, there is a possibility for larger differences in other datasets, such as data from cfDNA, RRBS, methyl capture, and single-cell protocols, that have higher rates of PCR duplicates. Therefore, future work is needed to investigate how the choice of duplicate marking tool impacts analyses, particularly for analyses related to rare events, where small changes in coverage due to differences in duplicate marking may impact experimental conclusions.

Previously, Bismark’s duplicate marking utility, deduplicate_bismark, was the only WGBS-aware tool. However, it suffered from several shortcomings, including its restriction to only Bismark-aligned data, its inability to handle streamed input, and its high memory usage. Dupsifter provides an aligner-agnostic way to mark duplicates from WGBS data. It allows users to mark duplicates during the alignment process through streaming, while also allowing users to mark duplicates in already aligned data without the requirement to stream the input. Finally, dupsifter improves upon the performance of deduplicate_bismark by marking duplicates faster and with significantly less memory.

## Supplementary Material

btad729_Supplementary_Data

## Data Availability

WGBS data used in the performance testing is available on the legacy Genomic Data Commons site under TCGA LUSC 2600. scXRBS data were downloaded from SRA with SRA accession IDs: SRR11711253 and SRR11711254. GM12878 data were downloaded from SRA with SRA accession IDs: SRR4235788 and SRR4235789.
