# Supplementary material for: Dupsifter: a lightweight duplicate marking tool for whole genome bisulfite sequencing
Source: Bioinformatics. 2023 Dec 13;39(12):btad729. doi: 10.1093/bioinformatics/btad729 (PMC10724848; doi:10.1093/bioinformatics/btad729)
Supplement: btad729_Supplementary_Data [file btad729_supplementary_data.pdf]

Dupsifter Supplementary Materials

Supplementary Figures

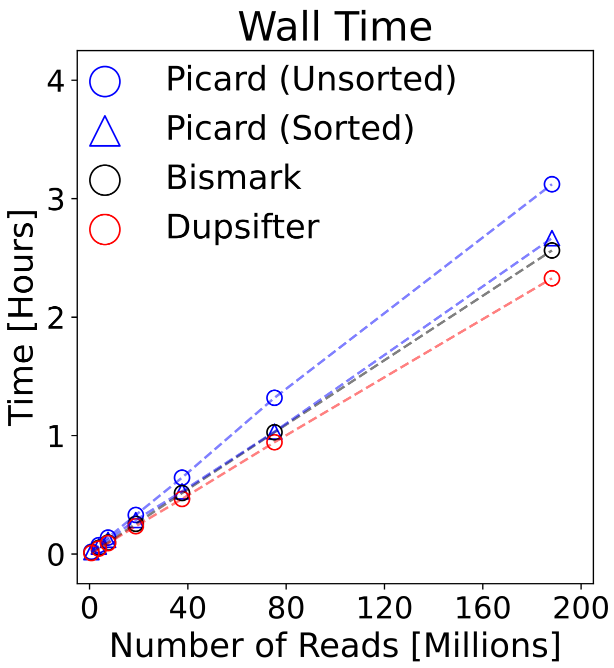

Supplemental Figure S1: **Wall time for dupsifter, Bismark's deduplicate_bismark, and Picard MarkDuplicates.** "Picard (Unsorted)" uses the BAM produced by Bismark, where the only guarantee about the sort order is that reads with the same name are grouped together. On the other hand, "Picard (Sorted)" uses samtools to coordinate sort the reads before marking duplicates with Picard.

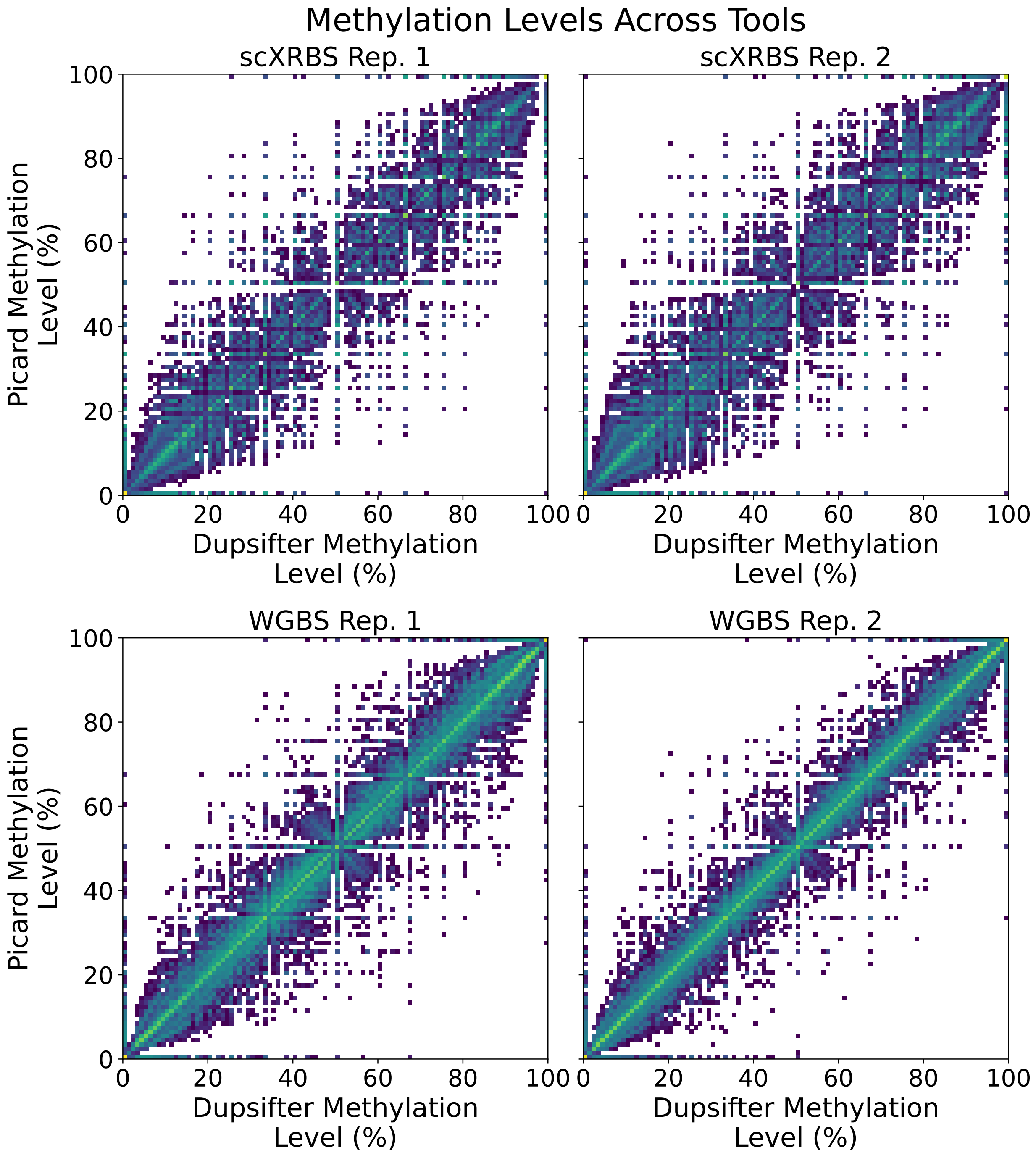

Supplemental Figure S2: **Comparison of methylation levels using dupsifter and Picard MarkDuplicates for the two scXRBS replicates (top) and two GM12878 WGBS replicates (bottom).**

Supplementary Methods

Performance Benchmarking

All performance benchmarking was performed on a server-class machine with 40 cores (2 Intel Xeon Gold 6138 CPUs running at 2.0 GHz per core) and 384 GB of memory.

Data used in the benchmarking are available on the legacy Genomic Data Commons site under TCGA LUSC 2600. Following download of the data, the FASTQ files were subsampled to 1, 5, 10, 25, 50, 100, and 250 million reads using seqtk (https://github.com/lh3/seqtk, version 1.3-r113-dirty):

```
seqtk sample reads.rX.fq.gz NNN | gzip > reads.NNN.rX.fq.gz
```

where NNN is the number of reads subsampled and X is either 1 or 2. Each subsampled set of FASTQ files was then aligned with Bismark [Krueger and Andrews (2011)] (version 0.24.0, Bowtie2 version 2.5.1):

```
bismark \
    --parallel 10 \
    --output_dir NNN \
    --temp_dir NNN \
    --samtools_path /path/to/samtools \
    --genome hg38 \
    -1 reads.NNN.r1.fq.gz \
    -2 reads. NNN.r2.fq.gz
```

Duplicate marking with dupsifter (version 1.2.0) was performed with:

```
dupsifter \
    --remove-dups \
    -o dupsifter.NNN.bam \
    hg38.fa \
    aligned.NNN.bam
```

For Bismark (version 0.24.0), duplicate marking was performed with:

```
deduplicate_bismark -p \
    --outfile bismark.NNN \
    aligned.NNN.bam
```

Picard MarkDuplicates (version 2.27.5, `http://broadinstitute.github.io/picard/`) was run in two different ways. First, for a closer comparison to dupsifter and deduplicate_bismark, which expect reads grouped by their name, Picard was run with the reads as they came out of Bismark (referred to as "unsorted"):

```
java -Xms8g -Xmx300g -Djava.io.tmpdir=/path/to/tmp \
    -jar $PICARD MarkDuplicates \
    I=aligned.NNN.bam \
    O=picard_unsorted_NNN.bam \
    M=picard_unsorted_NNN.stats \
    REMOVE_DUPLICATES=true \
    ASSUME_SORT_ORDER=queryname
```

In most cases, though, users coordinate sort their BAMs prior to using Picard; therefore, Picard was also run with a coordinate sorted BAM:

```
samtools sort -@ 8 -m 5G \
    -o picard_sorted.unmarked.NNN.bam \
```

```
    aligned.NNN.bam

java -Xms8g -Xmx300g -Djava.io.tmpdir=/path/to/tmp \
    -jar $PICARD MarkDuplicates \
    I=picard_sorted.unmarked.NNN.bam \
    O=picard_sorted_NNN.bam \
    M=picard_sorted_NNN.stats \
    REMOVE_DUPLICATES=true \
    ASSUME_SORT_ORDER=coordinate
```

In all cases, time and memory statistics were taken using GNU time (version 1.9). Duplicate marking for each of the four cases was run 5 times for each subsampled number of reads and the mean results reported.

Comparison of WGS and WGBS Duplicate Marking

In order to compare the reads marked as duplicates, two replicates (SRA accession IDs: SRR11711253 and SRR11711254) were used from the original scXRBS paper [Shareef et al. (2021)]. Raw FASTQs were downloaded from SRA for each replicate and then aligned with BISCUIT (version 1.2.1, `https://github.com/huishenlab/biscuit`):

```
biscuit align \
    -@ 30 \
    -b 1 \
    hg38.fa \
    SRA_1.fq.gz \
    SRA_2.fq.gz | \
samtools view \
    -@ 30 \
    -hbo SRA.name_sorted.bam -
```

where `SRA` is the SRA accession ID for each replicate.

After alignment, duplicates were marked with both dupsifter (in WGBS and WGS modes) and Picard MarkDuplicates:

```
# Dupsifter (WGBS)
dupsifter \
    -o SRA.wgbs.markdup.bam \
    -O SRA.wgbs.dupsifter.stats \
    hg38.fa \
    SRA.name_sorted.bam

# Dupsifter (WGS)
dupsifter \
    --wgs-only \
    -o SRA.wgs.markdup.bam \
    -O SRA.wgs.dupsifter.stats \
    hg38.fa \
    SRA.name_sorted.bam
```

```
# Picard MarkDuplicates
java -Xms8g -Xmx300g -Djava.io.tmpdir=/path/to/tmp \
    -jar $PICARD MarkDuplicates \
    I=SRA.name_sorted.bam \
    O=SRA.picard.markdup.bam \
    M=SRA.picard.stats \
    REMOVE_DUPLICATES=false \
    ASSUME_SORT_ORDER=queryname
```

In both dupsifter and Picard, both reads in a pair are marked as duplicates if they meet the qualifications, even in instances where one read in the pair is unmapped. For this reason, the duplicate rate is the number of read pairs marked as duplicates over the total number of read pairs (see Supplemental Table S1 for the number of read pairs in each replicate). For the differences between dupsifter in WGBS and either dupsifter in WGS mode or Picard MarkDuplicates, the number of read pairs where at least one read in the pair differed between the two tools was counted and then the percentage calculated relative to the total number of read pairs. The "total different" is the sum of the two combinations of duplicate versus non-duplicate.

Accession ID	Number of Read Pairs
SRR11711253	60,648,809
SRR11711254	80,318,302

Supplemental Table S1: **Number of read pairs in each replicate used in comparing duplicate marking with WGBS-aware and non-WGBS-aware tools.**

Methylation Level Analysis

To compare the relative impact of duplicate marking tool choice on methylation levels, we used two WGBS replicates of the well-characterized GM12878 cell line (SRA accession IDs: SRR4235788 and SRR4235789) [Dunham et al. (2012)], in addition to the two scXRBS samples described previously. FASTQs from each replicate were downloaded from SRA and trimmed using TrimGalore! (version 0.6.6 with cutadapt 4.1, `https://github.com/FelixKrueger/TrimGalore`) and subsampled to 500 million reads each. Reads were aligned with BISCUIT (version 1.2.1), duplicate marked with either dupsifter (version 1.2.0) or Picard MarkDuplicates (version 2.27.5), then methylation was extracted using BISCUIT:

```
# Align
biscuit align \
    -@ 30 \
    -b 1 \
    hg38.fa \
    SRA_1_val_1_500M.fq.gz \
    SRA_2_val_2_500M.fq.gz | \
```

```
samtools view \
    -@ 30 \
    -hbo SRA.name_sorted.bam -

# Duplicate mark
dupsifter \
    -o SRA.dupsifter.markdup.bam \
    -O SRA.dupsifter.stats \
    hg38.fa \
    SRA.name_sorted.bam

java -Xms8g -Xmx300g -Djava.io.tmpdir=/path/to/tmp \
    -jar $PICARD MarkDuplicates \
    I=SRA.name_sorted.bam \
    O=SRA.picard.markdup.bam \
    M=SRA.picard.stats \
    REMOVE_DUPLICATES=false \
    ASSUME_SORT_ORDER=queryname

# Sort and index
samtools sort \
    -@ 30 -m 5G \
    -o SRA.dupsifter.sorted.markdup.bam \
    SRA.dupsifter.markdup.bam
samtools index -@ 30 SRA.dupsifter.sorted.markdup.bam

samtools sort \
    -@ 30 -m 5G \
    -o SRA.picard.sorted.markdup.bam \
    SRA.picard.markdup.bam
samtools index -@ 30 SRA.picard.sorted.markdup.bam

# Methylation extraction
biscuit pileup \
    -@ 30 \
    hg38.fa \
    SRA.dupsifter.sorted.markdup.bam | \
biscuit vcf2bed - | \
biscuit mergecg -c hg38.fa - | \
awk 'BEGIN{FS=OFS="\t"} {print $1, $2+1, $3-1, $4, $5, $6}' | \
bgzip -c > SRA.dupsifter.cgmerge.cov.gz

biscuit pileup \
    -@ 30 \
    hg38.fa \
    SRA.picard.sorted.markdup.bam | \
biscuit vcf2bed - | \
```

```
biscuit mergecg -c hg38.fa - | \
awk 'BEGIN{FS=OFS="\t"} {print $1, $2+1, $3-1, $4, $5, $6}' | \
bgzip -c > SRA.picard.cgmerge.cov.gz
```

where `SRA` is the SRA accession ID for each replicate. Briefly, CG loci were only considered if there were at least 3 spanning reads and methylation was collapsed across strand before summarizing in the Bismark coverage file format.

Methylation for the scXRBS samples proceeded in a similar manner, with the one exception that they were summarized in the BISCUIT BED format (`biscuit mergecg hg38.fa - | bgzip > SRA.bed.gz`).

References

[Dunham et al. (2012)] Dunham,I. *et al.* (2012) An integrated encyclopedia of DNA elements in the human genome, *Nature*, **489**, 57–74, https://doi.org/10.1038/nature11247.

[Krueger and Andrews (2011)] Krueger,F. and Andrews,S.R. (2011) Bismark: a flexible aligner and methylation caller for Bisulfite-Seq applications, *Bioinformatics*, **27**, 1571—1572, https://doi.org/10.1093/bioinformatics/btr167.

[Shareef et al. (2021)] Shareef,S.J. *et al.* (2021) Extended-representation bisulfite sequencing of gene regulatory elements in multiplexed samples and single cells, *Nat Biotechnol*, **39**, 1086–1094, https://doi.org/10.1038/s41587-021-00910-x.
